# Resilience to emergencies and civil society organizations

**DOI:** 10.2471/BLT.22.289237

**Published:** 2023-03-01

**Authors:** Hanna Teräs, Nellie Kartoglu

**Affiliations:** a School of Pedagogical Innovations and Culture, Tampere University of Applied Sciences, Kuntokatu 3, 33520 Tampere, Finland.; b Community Readiness and Resilience Department, World Health Organization, Geneva, Switzerland.

Health and development stakeholders increasingly recognize the role of civil society organizations in contributing to building community resilience to public health emergencies.^1^ Civil society organizations play a central role in delivering services and spreading awareness in communities, supporting the implementation of campaigns, and binding public and private outreach and development activities.^2^ These organizations contribute to social innovation;^3^ if empowered, they could become multipliers of knowledge.^4^

Between 2020 and 2022, with the support of the COVID-19 Solidarity Response Fund, the World Health Organization (WHO) engaged 54 civil society organizations in 40 countries to mitigate the impact of the coronavirus disease 2019 (COVID-19) pandemic in the communities they serve. Over 80 million people in vulnerable settings benefited from this first-time direct partnership at the community level. This collaboration was based on joint planning of interventions in alignment with national and local COVID-19 response strategies.^5^ The partnership has leveraged life-changing impact in many communities – from enabling sustainable livelihoods to including vulnerable groups in national emergency preparedness and response plans. The impact of these interventions demonstrates the crucial role of local civil society organizations in service delivery, especially in communities where governments have limited or no access. Participatory community structures engaging community representatives and local authorities contribute to policy dialogue on inclusion, and demonstrate the need to systematically involve grassroots civil society organizations in decision-making, planning, implementation, monitoring and shared accountability regarding policies that affect the health and well-being of their communities.

Researchers recognize that providing these organizations with resources and skills for intranational and international cooperation is a priority in battling the effects of public health emergencies.^6^ Moreover, researchers have identified a growing need for organizations to obtain clear, relevant and easily accessible information concerning public health emergencies.^7^ Such studies largely see the learning of volunteers associated with civil society organizations and nongovernmental organizations as peripheral to volunteer work. Research and development have focused on the volunteers’ work, overlooking their learning.^8^ However, volunteers learn constantly by acquiring skills from the work they do.^9^ Consequently, a wealth of knowledge exists in communities and civil societies, much of which goes unnoticed, unleveraged and unshared.

Acknowledging the rising need for streaming up community expertise for the collaborative construction of knowledge, WHO has set up an informal online learning platform – the Knowledge Sharing Platform.^10^ The platform aspires to contribute to building resilient communities by engaging civil society organizations in learning through sharing experiences, best practices, solutions to health challenges and lessons learnt through grassroot involvement. The platform aims to make the knowledge and experience of these organizations visible and shared in an upstream manner. The platform also offers an opportunity for WHO and other health and development partners to better understand community needs. Better understanding of community needs helps health and development partners and governments to streamline solutions to evidence-based decision-making, and guides community-centred and community-led public health policies.

Developed on design principles derived from theories of andragogy (the science and practice of adult education), informal and self-directed learning, as well as online communities of practice, the platform is not an online learning platform or programme, but a space that encourages and values user expertise and contribution, and allows for different ways of using and interacting with content and features.^9^^,^^11^^,^^12^ Learning on the platform takes place through user-generated case studies, examples of participatory community structures, voices from communities and perspectives of thought leaders – all of which is supported by distributing curated information in the knowledge bank and moderated online discussions.

The platform’s piloting phase in the second half of 2022 brought together a group of civil society organizations from different parts of the world to test and evaluate the platform. Evaluation data were collected through a focus group webinar and an online questionnaire, and the results informed the further improvement of the platform. In open answers, the respondents indicated that such a platform is useful for several purposes – sharing and showcasing one’s work, learning from others, communicating with others, acquiring or contributing to relevant information on health promotion beyond emergencies, and advocating for community-centred health approaches.

The results from the platform’s evaluation testify to the importance of informal knowledge sharing and learning opportunities for community stakeholders, so that community experiences are included in formulating policies that affect a community’s well-being. Creating informal collaborative knowledge sharing spaces for community stakeholders can be one of the most efficient ways to enhance community engagement for building community readiness and resilience to public health emergencies.
